# Monetary costs of agitation in older adults with Alzheimer's disease in the UK: prospective cohort study

**DOI:** 10.1136/bmjopen-2014-007382

**Published:** 2015-03-13

**Authors:** Stephen Morris, Nishma Patel, Gianluca Baio, Lynsey Kelly, Elanor Lewis-Holmes, Rumana Z Omar, Cornelius Katona, Claudia Cooper, Gill Livingston

**Affiliations:** 1 Department of Applied Health Research, University College London, London, UK; 2 Department of Statistical Science and PRIMENT Clinical Trials Unit, University College London, London, UK; 3 Division of Psychiatry, University College London, London, UK

**Keywords:** HEALTH ECONOMICS, MENTAL HEALTH

## Abstract

**Objective:**

While nearly half of all people with Alzheimer's disease (AD) have agitation symptoms every month, little is known about the costs of agitation in AD. We calculated the monetary costs associated with agitation in older adults with AD in the UK from a National Health Service and personal social services perspective.

**Design:**

Prospective cohort study.

**Setting:**

London and the South East Region of the UK (LASER-AD study).

**Participants:**

224 people with AD recruited between July 2002 and January 2003 and followed up for 54 months.

**Primary and secondary outcome measures:**

The primary outcome was health and social care costs, including accommodation costs and costs of contacts with health and social care services. Agitation was assessed using the Neuropsychiatric Inventory (NPI) agitation score.

**Results:**

After adjustment, health and social care costs varied significantly by agitation, from £29 000 over a 1 year period with no agitation symptoms (NPI agitation score=0) to £57 000 at the most severe levels of agitation (NPI agitation score=12; p=0.01). The mean excess cost associated with agitation per person with AD was £4091 a year, accounting for 12% of the health and social care costs of AD in our data, and equating to £2 billion a year across all people with AD in the UK.

**Conclusions:**

Agitation in people with AD represents a substantial monetary burden over and above the costs associated with cognitive impairment.

**Strengths and limitations of this study**

- This study used detailed, prospectively collected health and social care resource use data plus data on frequency and severity of agitation symptoms over a 54-month period to calculate the costs of agitation in people with Alzheimer's disease (AD).
- There is no previous evidence about the cost of agitation in AD, even though nearly half of all people with AD have agitation symptoms every month; this study calculated that the mean excess cost associated with agitation per person with AD was £4091 a year.
- A limitation of the study is that it is based on a relatively small data set of 224 people, recruited to be representative of those with AD between July 2002 and January 2003 and followed up to 54 months.
- We did not include the costs of informal care; these data were not collected and UK guidelines for undertaking economic evaluations recommend taking a health and social care perspective when measuring costs.

## Introduction

The monetary cost of dementia is huge, with an estimated global burden in 2010 of US$604 billion incurred by health (16% of the total) and social care (42%) services and informal care (42%).^1^ Around 70% of worldwide costs occur in North America and Western Europe^1^; estimates for the UK show that the total monetary cost of dementia in 2014 was £26 billion.^2^ Alzheimer’s disease (AD) is the most common form of dementia, accounting for around 62% of cases.^2^

Nearly half of all people with AD have agitation symptoms every month.^3^ These are positively correlated with institutionalisation,^4^ pharmacological treatment and use of medical services,^3^ but there is no evidence on the costs of agitation in people with AD.^5^ ^6^ The aim of this paper is to calculate the monetary costs associated with agitation in AD.

## Methods

### Participants

We calculated National Health Service (NHS) and personal social services (PSS) costs associated with different levels of agitation using data from a naturalistic prospective cohort study of people with AD, covering the London and the South East Region of the UK (LASER-AD study).^3^ ^7–9^ Two hundred and twenty-four people were recruited between July 2002 and January 2003 and followed up to 54 months. The cohort was purposively and prospectively recruited, using overall figures from a review of the epidemiology of AD, to be a representative sample of people with AD in terms of sex, living setting and severity of cognitive impairment in the community.^9^ Participants and their carers were approached through local community mental health teams, dementia specialist nurses, the voluntary sector, memory clinics, nursing and residential homes, day hospitals, day centres and inpatient units. Written informed consent was obtained from all carers. Where the person with AD lacked capacity to consent, the study only proceeded if the carer consented and thought the person they cared for would have agreed to participate if they could. Measures were collected at baseline, and 18, 30, 42 and 54 months after baseline. Data were obtained from interviews with the patients with AD and their carers, carried out at a place of their choice. They were conducted by trained, experienced health professionals, and were terminated if the interviewee became distressed or appeared to want to stop.

### Measures

The Neuropsychiatric Inventory (NPI) uses responses from caregivers in a structured interview format to assess 10 behavioural domains (delusions, hallucinations, agitation, dysphoria, anxiety, apathy, irritability, euphoria, disinhibition, aberrant motor behaviour);^10^ two additional domains (night-time behavioural disturbance, appetite/weight changes) are commonly added, giving 12 domains in total.^11^ Within each domain, behaviours are rated by caregivers in terms of frequency (1=occasionally—less than once per week, 2=often—about once per week, 3=frequently—several times per week but less than every day, 4=very frequently—once or more per day) and severity (1=mild, 2=moderate, 3=severe). A score for each domain is calculated as the product of the frequency and severity scores, giving nine possible values (0, 1, 2, 3, 4, 6, 8, 9, 12), including no symptoms (=0). A score >3 on any domain is usually regarded as clinically significant.^3^ ^12–14^ A total NPI score is obtained by summing all the individual domain scores across the 12 domains, giving a range from 0 to 144. Agitation was assessed at each time point in the LASER-AD study using the agitation domain of the NPI, with higher values indicating more severe levels of agitation.

### Resource use and costing

Resource use was measured using the Client Service Receipt Inventory, amended for use with older people^15^ and collected from participant responses and caregiver reports for the previous 3 months at each time point. This incorporated information on where the person was living (at home, residential respite care, day respite care, residential care home (where staff typically do not have nursing qualifications), nursing care home, sheltered housing with a warden in the premises during the day, hospital awaiting placement), and their contacts with health and social care services (general practitioner (GP), practice nurse at the GP surgery, district nurse at the person's home, dietician, community psychiatric nurse, home help, meals on wheels, physiotherapist, chiropodist, optician, dentist, audiologist, psychologist, psychiatrist, day centre, hospital outpatient visits and inpatient stays). We did not include the costs of informal care—these data were not collected; we focused on health and social care costs, which is the costing perspective recommended in economic analyses in the UK.^16^ We applied unit costs from routine sources^17^ ^18^ in 2011 UK£ and calculated 3-month costs for each participant at each follow-up point. Three-month costs were multiplied by 4 to create 12-month figures.

### Statistical analyses

We calculated unadjusted mean and median 12-month costs by NPI agitation score (≤3, >3) and examined between-group differences using one-way analysis of variance (ANOVA), and the Mann-Whitney two-sample test. We examined associations between NPI agitation score and demographic variables, coexisting conditions and cognitive impairment using χ^2^ tests. We calculated descriptive statistics for caregivers, who assessed behaviours using the NPI and recorded resource use. We calculated unadjusted mean and median 12-month costs by individual NPI agitation score and tested for significant differences using one-way ANOVA with Bonferroni correction for multiple tests, and χ^2^ tests on the equality of medians. Use of health and social services among people with AD who are agitated may be affected by the extent of cognitive decline, demographic factors and comorbidities; to isolate the costs associated with agitation, we ran analyses adjusting for these factors. To account for skewness of the cost data, we used a generalised linear model with γ family and log link,^19^ adjusting for gender and age (using five 10-year bands) at baseline, marital status (6 categories), ethnic group (9 categories), highest level of education (5 categories), previous employment (9 categories), rurality (2 categories), coexisting conditions (diabetes, stroke, hypertension, heart disease), total NPI agitation score (in our data the range of scores was 0–82 with 66 unique values; we included categorical indicators for each score, including 66 categories in total), cognitive impairment (measured using the Mini-Mental State Examination;^20^ 31 categories), and follow-up point (baseline, 18, 30, 42, 54 months). We also considered using log Normal, Gaussian, inverse Gaussian and negative binomial distributions, but the γ model gave the best fit in terms of residual plots and the Akaike Information Criterion. We adjusted for clustering for repeated measures by participant using clustered sandwich estimators for the SE that allowed for intragroup correlation within participants. We predicted 12-month health and social care mean costs by NPI agitation score, controlling for the covariates. The differences in adjusted means were tested using Wald tests. In intervention studies, outcomes are sometimes measured in terms of change in NPI agitation scores, so we re-ran the analyses including NPI agitation scores as a linear term rather than categorical indicators.

### Excess costs associated with agitation

We combined the adjusted annual costs per person at different levels of NPI agitation score with prevalence rates in the LASER-AD study to calculate the annual expected cost per person with AD based on the per cent with each NPI agitation score. From this, we subtracted the adjusted annual costs per person with no agitation symptoms (NPI score=0) to estimate the mean excess costs associated with agitation per person each year. We also calculated UK-specific excess costs of agitation based on the prevalence of AD in the UK.

## Results

### Baseline characteristics of caregivers

The mean age of caregivers (SD) was 63 years (14 years). Most caregivers were female (69%), married (69%), had no children living at home (75%) and were living with the person with AD (56%; see online supplementary table S2). The modal relationship to the person with AD was ‘Child’ (35%).

### Health and social care costs associated with agitation

Of the 224 participants in the LASER-AD study, 111 had died by 54 months; our data set had 695 data points (person follow-ups). We applied unit costs to the resource use data in the LASER-AD study (see online supplementary table S1). Unadjusted mean (SD) per capita annual costs for participants with NPI agitation score ≤3 and >3 were £27 752 (£38 413) and £38 910 (£46 150; p<0.001, table 1). Median (IQR) values were £24 796 (£3512–£38 656) and £28 492 (£11 680–£40 164; p=0.001). Cost data were highly skewed (see online supplementary figure S1). The mode and median NPI agitation score were 0 and 1, respectively (table 2). Table 1 shows the per cent of the sample with different demographic variables, coexisting conditions and cognitive impairment by NPI agitation score. People with agitation scores >3 had a higher mean and median total NPI score, were more likely to be single and divorced and less likely to be married, less likely to be educated to secondary level and more likely to be educated to tertiary level, more likely to have heart disease, and more likely to have severe cognitive impairment (p<0.05).

**Table 1:** Descriptive statistics of sample by agitation symptoms

	NPI agitation score ≤3 (N=493)	NPI agitation score >3 (N=202)	p Value
	Mean and median		
Per capita annual cost*			
Mean (SD)	27 752 (38 413)	38 910 (46 150)	<0.001
Median (IQR)	24 796 (3512–38 656)	28 492 (11 680–40 164)	0.001
Total NPI score			
Mean (SD)	14 (11)	32 (16)	<0.001
Median (IQR)	12 (6–19)	29 (21–42)	<0.001
Per cent			
Gender			
Male	27.8	28.7	0.80
Female	72.2	71.3	
Age category (years)			
50–59	1.4	1.5	
60–69	8.1	5.9	
70–79	33.7	45.5	0.06
80–89	46.9	37.6	
90–99	9.9	9.4	
Marital status			
Single	4.9	8.4	
Married	40.6	34.7	
Separated	1.4	0.5	0.01
Divorced	2.4	5.0	
Widower	50.5	49.5	
Other	0.2	2.0	
Ethnic group			
White British	78.5	75.3	
White Irish	7.1	8.9	
White other	9.5	8.9	
Greek	0.4	1.0	
Black Caribbean	2.6	3.0	0.65
Black other	0.4	1.5	
Indian	0.2	0.0	
Pakistani	0.4	0.0	
Other	0.8	1.5	
Highest level of education			
Primary	2.6	4.5	
Secondary	82.2	69.3	
Tertiary	9.9	13.9	0.002
Other	0.6	1.0	
Not known	4.7	11.4	
Previous employment			
Manager/administrator	6.7	4.0	
Professional	11.0	5.9	
Associate professional	1.8	2.0	
Clerical worker/secretary	18.9	17.8	
Skilled labourer	18.5	23.8	0.23
Services/sales	15.2	15.4	
Factory worker	11.2	8.9	
Other	15.6	20.3	
Don’t know	0.2	0.0	
Rurality			
Urban	90.7	90.6	0.98
Rural	9.3	9.4	
Diabetes			
No	89.9	95.1	
Yes: IDDM	1.6	0.0	0.09
Yes: NIDDM—medically controlled	7.3	4.0	
Yes: NIDDM—diet controlled	1.2	1.0	
Stroke			
No	90.7	92.1	0.55
Yes	9.3	7.9	
Hypertension			
No	63.5	69.8	0.11
Yes	36.5	30.2	
Heart disease			
No	95.9	91.6	0.02
Yes	4.1	8.4	
Cognitive impairment			
Mild (MMSE 21–30)	26.4	8.9	
Moderate (MMSE 10–20)	37.7	28.2	<0.001
Severe (MMSE ≤9)	35.9	62.9	

*2011 UK£.

IDDM, insulin-dependent diabetes mellitus; MMSE, Mini-Mental State Examination; NIDDM, non-insulin-dependent diabetes mellitus; NPI, Neuropsychiatric Inventory.

**Table 2:** Association between agitation symptoms and per capita annual cost*: unadjusted and adjusted analyses (N=695)

NPI agitation score	Number (%)	Mean (SD)	Median (IQR)	Adjusted mean (95% CI)†
0	314 (45.2)	28 218 (43 332)	13 962 (3048–36 444)	28 983 (24 364 to 33 603)
1	68 (9.8)	22 596 (24 266)	22 352 (2666–32 149)	43 910 (30 618 to 57 203)
2	60 (8.6)	29 544 (34 427)	27 328 (8546–39 088)	31 196 (22 903 to 39 490)
3	51 (7.1)	29 653 (23 081)	28 216 (9089–39 076)	35 120 (25 592 to 44 648)
4	60 (8.6)	27 909 (23 353)	27 566 (6796–38 728)	35 458 (26 843 to 44 074)
6	57 (8.2)	35 324 (40 889)	27 648 (9720–39 028)	25 138 (17 918 to 32 358)
8	45 (6.5)	42 289 (48 695)	31 076 (23 616–42 532)	36 568 (25 590 to 47 545)
9	12 (1.7)	46 589 (41 302)	39 388 (18 017–64 726)	38 568 (11 867 to 65 269)
12	28 (4.0)	61 064 (76 070)	36 794 (27 126–44 468)	57 023 (31 861 to 82 186)
p Value		0.001	<0.001	0.01

*2011 UK£.

†Controls are included for age, gender, marital status, ethnic group, highest level of education, previous employment, rurality, coexisting conditions (diabetes, stroke, hypertension, heart disease), total NPI score, cognitive impairment (MMSE) and follow-up.

MMSE, Mini-Mental State Examination; NPI, Neuropsychiatric Inventory.

Unadjusted mean and median costs increased with agitation score (p≤0.001; table 2).

**Figure 1:**
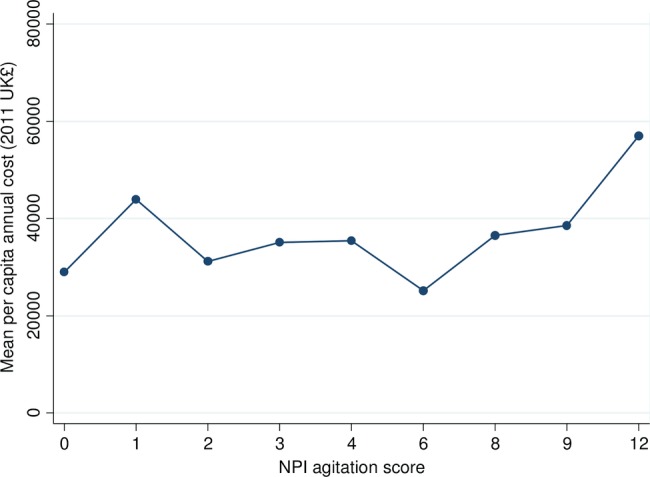
Adjusted^a^ mean per capita annual cost^b^ by agitation symptoms (N=695). ^a^Controls are included for age, gender, marital status, ethnic group, highest level of education, previous employment, rurality, coexisting conditions (diabetes, stroke, hypertension, heart disease), total NPI score, cognitive impairment (MMSE) and follow-up. ^b^2011 UK£. NPI, Neuropsychiatric Inventory; MMSE, Mini-Mental State Examination.

After adjusting for demographic variables, coexisting conditions, cognitive impairment, follow-up and individual clustering for repeated measures, mean costs varied by NPI agitation scores, from £29 000 over a 12-month period with no agitation symptoms (NPI agitation score=0) up to £57 000 at the most severe levels of agitation (NPI agitation score=12; p=0.01, table 2 and figure 1). Costs also varied significantly by age and gender, marital status, ethnic group, highest level of education, total NPI score and cognitive impairment (p<0.05, see online supplementary table S3).

When we reran the model including NPI agitation scores as a linear term rather than categorical indicators, we found that a one-unit increase in NPI agitation scores was associated with a £1736 increase in costs per patient over a 12-month period (95% CI £644 to £2807, p=0.001) in an unadjusted model, and £1064 (95% CI −£34 to £2162, p=0.058) when adjusting for the covariates.

### Excess costs associated with agitation in the UK

The adjusted annual expected cost per person with AD based on the per cent with each NPI agitation score in our sample was £33 075 and the adjusted annual costs per person with no agitation symptoms was £28 983 (see online supplementary table S4). Hence, the excess cost associated with agitation per person with AD was £4091 a year. This suggests that on average agitation accounts for 12% (£4091/£33 075) of the health and social care costs of AD each year. In the UK, there are 800 000 people with dementia and around 62% of cases are accounted for by AD.^2^ The expected excess cost associated with agitation in people with AD is therefore £2.0 billion a year (£4091×800 000×0.62).

## Discussion

### Principal findings

Among people with AD, health and social care costs varied significantly by the level of agitation, from £29 000 over a 12-month period in people with no agitation symptoms up to around £57 000 at the most severe levels of agitation. On average, agitation symptoms account for 12% of the health and social care costs of AD. The excess cost associated with agitation was £2 billion a year across all people with AD in the UK.

### Strengths and weaknesses

Our analysis is based on a unique data set containing very detailed information on frequency and severity of agitation symptoms and use of health and social care services over a 54-month time period. The data also include a range of demographic variables, coexisting conditions and cognitive impairment that can be included to isolate the costs associated with agitation.

With regard to limitations, the data set is relatively small, containing 224 people with AD. Given the large number of covariates included in our models, the fact that agitation is a significant predictor of costs suggests that the relationship is a strong one. Participants were recruited between July 2002 and January 2003 and followed up to 54 months; hence, the data are relatively old and the prevalence of agitations symptoms among people with AD may have changed over time. In addition, management practices might have changed over time. For example, in 2006, the National Institute for Health and Care Excellence in England first published guidance on the use of medications and treatments for AD; this was amended in 2007 and 2009, and new updated guidance that recommended extending the use of drug treatment in AD was issued in 2011.^21^ Prescribing practices have changed over time with a marked reduction in antipsychotic drug use in people with dementia: the mean prevalence of antipsychotic use on diagnosis of dementia fell in the UK from 19.9% in 1995 to 7.4% in 2011.^22^ While participants were selected to be representative of patients with AD, they were recruited from one geographical area, potentially limiting generalisability. We did not include the costs of informal care, though these have been estimated to account for a substantial proportion of the total costs of dementia.^1^ ^2^ ^23^ These data were not collected in the LASER-AD study. UK guidelines for undertaking economic evaluations recommend taking a health and social care perspective when measuring costs.^16^

### Comparison with other studies

Several studies have evaluated the relationship between behavioural symptoms and costs of care associated with AD, but none have specifically evaluated the monetary cost of agitation in AD. The studies evaluating the impact of behavioural symptoms on costs of care have tended to find a positive relationship. For example, using data from the USA on 128 patients with AD Murman *et al*^24^ found that after controlling for cognitive impairment and comorbidities behavioural symptoms measured using the NPI significantly increased total direct costs (healthcare costs plus informal care costs): a one-point increase in total NPI score was associated with an annual increase of between US$247 and US$409 in total direct costs, depending on the value of unpaid caregiving. Gustavsson *et al* found that in a sample of 1222 patients with AD from Spain, Sweden, the UK, and the USA, there was a significant relationship between behavioural symptoms measured using total NPI score and cost of health and social care among people living in the community after controlling for ability to perform activities of daily living and cognitive impairment: a one-point increase in total NPI score was associated with a 1% increase in health and social care costs. Among people living in residential care, a one-point increase in total NPI score was associated with a 1.6% increase in costs of care in the USA only.^25^ Using data for 272 patients with AD attending six memory clinics in Sweden, Denmark, Norway and Finland, Jönsson and Eriksdotter Jönhagen^26^ found that total NPI score was significantly associated with health and social care plus informal care costs: after controlling for cognitive impairment, years since diagnosis of AD and comorbidities costs were calculated to increase by 8% for each one-point increase in total NPI score.

### Implications for clinicians and policymakers

People with AD who are agitated are substantial users of health and social care services, suggesting that effective measures to reduce agitation would reduce the burden on these services, as well as providing health benefits to people with AD and their carers. Reducing agitation could be cost-effective and, in addition, bring considerable cost savings, which should be compared against the cost of interventions.

### Further research

Health economic analyses of interventions for reducing agitation in AD incorporated into clinical trials are needed. Such analyses should evaluate the impact of interventions using final outcomes such as quality-adjusted life years, for example, using new approaches based on the DEMQOL system,^27^ ^28^ where cost-effectiveness thresholds have been identified.^16^ They should also include comprehensive cost analyses, including health and social care costs associated with managing agitation as well as intervention costs, and be conducted over sufficiently long time horizons to measure the full costs and benefits.
